# Prevention of depression and anxiety in adolescents: A randomized controlled trial testing the efficacy and mechanisms of Internet-based self-help problem-solving therapy

**DOI:** 10.1186/1745-6215-10-93

**Published:** 2009-10-12

**Authors:** Willemijn Hoek, Josien Schuurmans, Hans M Koot, Pim Cuijpers

**Affiliations:** 1 Department of Clinical Psychology, VU University, Amsterdam, the Netherlands; 2 Department of Developmental Psychology, VU University, Amsterdam, the Netherlands; 3 EMGO Institute, VU University Medical Centre, Amsterdam, the Netherlands

## Abstract

**Background:**

Even though depression and anxiety are highly prevalent in adolescence, youngsters are not inclined to seek help in regular healthcare. Therapy through the Internet, however, has been found to appeal strongly to young people. The main aim of the present study is to examine the efficacy of preventive Internet-based guided self-help problem-solving therapy with adolescents reporting depressive and anxiety symptoms. A secondary objective is to test potential mediating and moderating variables in order to gain insight into how the intervention works and for whom it works best.

**Methods/design:**

This study is a randomized controlled trial with an intervention condition group and a wait-list control group. The intervention condition group receives Internet-based self-help problem-solving therapy. Support is provided by a professional and delivered through email. Participants in the wait-list control group receive the intervention four months later. The study population consists of adolescents (12-18-year-olds) from the general population who report mild to moderate depressive and/or anxiety symptoms and are willing to complete a self-help course. Primary outcomes are symptoms of depression and anxiety. Secondary outcomes are quality of life, social anxiety, and cost-effectiveness. The following variables are examined for their moderating role: demographics, motivation, treatment credibility and expectancy, externalizing behaviour, perceived social support from parents and friends, substance use, the experience of important life events, physical activity, the quality of the therapeutic alliance, and satisfaction. Mediator variables include problem-solving skills, worrying, mastery, and self-esteem. Data are collected at baseline and at 3 weeks, 5 weeks, 4 months, 8 months, and 12 months after baseline. Both intention-to-treat and completer analyses will be conducted.

**Discussion:**

This study evaluates the efficacy and mechanisms of Internet-based problem-solving therapy for adolescents. If Internet-based problem-solving therapy is shown to reduce depressive and anxiety symptoms in adolescents, the implication is to implement the intervention in clinical practice. Strengths and limitations of the study are discussed.

**Trial registration:**

Netherlands Trial Register NTR1322

## Background

Depression and anxiety disorders are the most prevalent mental disorders in adolescence, with lifetime prevalences between 17% and 28% by the age of 18 [1-3]. Furthermore, adolescent depressive and anxiety disorders are associated with decreased levels of functioning in various areas. Symptoms of depression and anxiety are even more common and are also related to various maladaptive outcomes. Specifically, low peer contact and peer rejection (e.g., [4]), social problem-solving deficits [5], a negative self-image [6,7], low perceived quality of social support [8,9], substance abuse (e.g., [10]), behavioural problems [11], poor parent-child relationships (e.g., [12]), and learning disabilities [13], to name a few, are all reported to be associated with symptoms of depression and anxiety. Depression and anxiety disorders also tend to have their first onset in adolescence and often show a chronic course with a high risk of relapse, which makes it important to prevent or postpone the onset of these mental health problems [14,15].

Despite the high incidence of common mental health problems in adolescence, teenagers rarely seek professional help for their emotional problems through regular healthcare services. The Internet, however, offers the possibility to access this large group of untreated adolescents for adequate care: it has low threshold acceptability, is used frequently by young people, and reduces objections like lack of willingness to talk to a stranger about personal problems and fear of stigma [16]. A recent study among a community sample of adolescents, found the Internet to be an acceptable medium through which they tend to seek help [17].

For adults, preventive self-help interventions have been offered through the Internet and proven to be effective in reducing symptoms of depression and anxiety (e.g., [18-21]). Self-help can be described as a standardized psychological treatment in which a patient can help himself, and whereby possible supervision by a therapist is merely supporting and facilitating. Internet-based self-help is attractive for several reasons. Besides the obvious advantages of the Internet, such as its low threshold acceptability and reduction of fear of stigma, the intervention does not require the use of extensively trained therapists. Therefore, self-help through the Internet constitutes a potentially cost-efficient and effective way to appropriately treat large groups of individuals [22]. In this manner, waiting lists may be reduced, travelling time is saved, and patients can work at their own pace [23]. Guided Internet self-help interventions have been found to be as effective as face-to-face treatments, with somewhat larger effects and lower dropout rates than unguided self-help interventions [24].

Most web-based self-help interventions for psychological problems have been developed for treating (symptoms of) specific disorders like depression or a specific anxiety disorder. Face-to-face problem-solving therapy (PST), on the other hand, has been found to be effective in a variety of problem areas [25,26]. A new preventive self-help intervention based on problem-solving was consequently developed for application through the Internet [27]. Bowman and colleagues' [28,29] Self-Examination Therapy (SET) ranks as the general framework for this intervention. The intervention was found to be successful in reducing adults' symptoms of depression, anxiety, and work-related stress [27].

While Internet-based self-help is effective in adults, it is still unknown whether these interventions also work for adolescents. Also, little is known with regard to the mechanisms underlying change in this form of treatment and potential predictors of treatment effect. For the current study, the Dutch Internet-based PST intervention [27] was adapted for use with adolescents. The PST intervention is targeted at indicated prevention, for those with subsyndromal levels of depression and/or anxiety. Subclinical manifestations are the best predictors of the onset of full-blown disorders [30]. Therefore, our Internet intervention is intended to reduce mild to moderate symptoms of depression and anxiety, thereby preventing or postponing the occurrence of depressive and anxiety disorders. We expect PST to be well suited for 12 to 18-year-olds because of its straightforward nature and the fact that it does not require complex skills or understanding of intrapersonal processes. As PST focuses on improving coping skills for problems and stressful events, it also links up nicely with the challenging and stressful phase of adolescence; the dynamic period in which people encounter and attempt to resolve many developmental challenges, including strengthening and expanding self-concepts, forming stable intimate relationships, making school and career decisions, and achieving a certain level of autonomy [31,32].

In the present study, we examine the effects of an Internet-based guided self-help intervention (PST) for adolescents reporting mild to moderate symptoms of depression and/or anxiety compared to a wait-list control group. Potential moderating and mediating variables are investigated in order to identify predictors of treatment effect and to evaluate potential underlying mechanisms of change. Moderating variables are explored, i.e., demographics, motivation, treatment credibility and expectancy, externalizing behaviour, peer and parental perceived social support, substance use, the experience of life events, physical activity, the working alliance between participant and coach, and satisfaction. Problem-solving abilities, mastery, self-esteem and ruminative responses are investigated for their possible mediating effect.

## Methods

### Study design

This study is a randomized controlled trial with two groups: the Internet-based self-help intervention group (PST) and a wait-list control group (WL). The study protocol has been approved by the Medical Ethics Committee of the VU University Medical Center.

### Inclusion and exclusion criteria

Adolescents (12 to 18-year-olds) with mild to moderate depressive and/or anxiety symptoms who are willing to participate in a self-help course are eligible for this study. Inclusion criteria are: sufficient knowledge of the Dutch language, access to Internet, and having an email address. Exclusion criteria are: absence of parental permission, already receiving treatment for mental health problems, the presence of severe depressive symptoms (defined as a score above 40 on the Centre for Epidemiologic Studies Depression scale; CES-D), severe anxiety symptoms (indicated by a score above 14 on the anxiety subscale of the Hospital Anxiety and Depression Scale; HADS-A), and/or prominent suicide ideation (indicated by a score above 1 on the suicide item of the Beck Depression Inventory-II; BDI-II).

### Procedure

**Figure 1:**
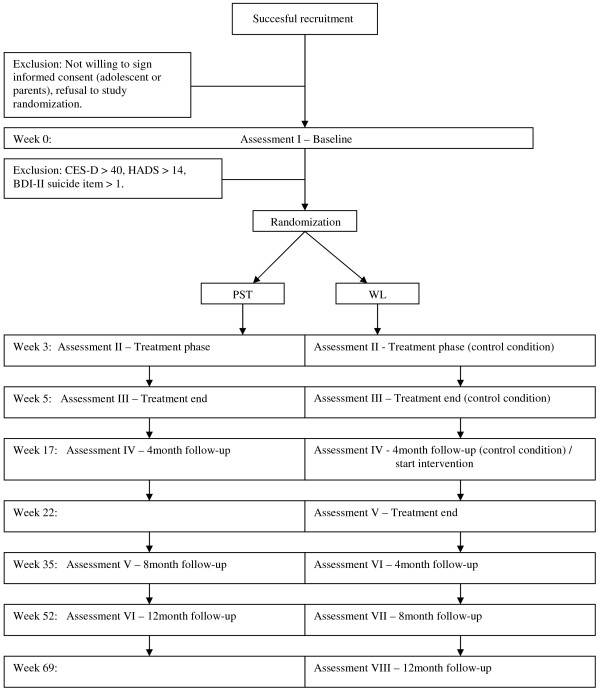
**Research procedure**.

Participants are recruited through banners and advertisements on the Internet, advertisements in magazines, referral by school-doctors, through brochures and posters in schools, and through information to parents who are treated in mental health care institutions for anxiety and depression. When signing in on the website, subjects receive a brochure and an informed consent form by email. In the brochure, information about the study's procedure and intervention is provided, and requirements for participation and parental consent are carefully explained. After application by the adolescent via email, parents receive a brochure and informed consent form by post. After receiving signed informed consent from both child and parents, participants receive an email with a link to the baseline questionnaire. Subjects with a score of 41 or higher on the CES-D are excluded. They receive a telephone call in which they are advised to consult their general practitioner. Their parents are also informed by telephone. The same procedure is followed in cases of a score of 15 or higher on the anxiety subscale of the HADS and/or a score of 2 or higher on the BDI-II suicide item. Eligible adolescents are randomized to either the intervention or the wait-list control condition, and are informed about the randomization outcome by email. Their parents also receive this email. Depression and anxiety subscales of the National Institute of Mental Health Diagnostic Interview Schedule for Children (NIMH-DISC IV) are subsequently conducted by telephone. Within two weeks after the baseline measurements, the intervention starts. Assessments take place during the treatment period, at treatment termination, and 4, 8, and 12 months after the start of the intervention. Figure 1 depicts the different stages of the research procedure. Table 1 provides an overview of the measurement instruments used at each assessment.

**Table 1:** Instruments at different assessment points.

	**Baseline**	**Treatment phase**	**Treatment end**	**4-month follow-up**	**8-month follow-up**	**12-month follow-up**
NIMH-DISC	X					
BDI-II	X	X	X	X	X	X
CES-D	X	X	X	X	X	X
HADS	X	X	X	X	X	X
PedsQL	X		X			X
SAS-A	X		X			X
Demographics	X					
Motivation	X					
CEQ	X					
WAI - SF			X			
CSQ-8			X			
YSR	X		X			
SSS-A	X		X			
Substance use	X		X			
LEQ-S	X			X		X
GS	X		X			
RSES	X	X	X			X
PSWQ-C	X	X	X			
Mastery Scale	X	X	X			
CISS	X	X	X			
TiC-P	X			X		

NIMH-DISC: *the National Institute of Mental Health Diagnostic Interview Schedule for Children*, BDI-II: *Beck Depression Inventory-II*, CES-D: *Centre for Epidemiological Studies Depression scale*, HADS: *Hospital Anxiety and Depression Scale*, PedsQL: *the Pediatric Quality of Life Inventory*, SAS-A: *Social Anxiety Scale for Adolescents*, CEQ: *Credibility/Expectancy Questionnaire*, WAI - SF: *Working Alliance Inventory, Short Form*, CSQ-8: *Client Satisfaction Questionnaire 8*, YSR: *Youth Self Report*, SSS-A: *Social Support Scale for Adolescents*, LEQ-S: *Life Events Questionnaire, Short form*, GS: *Godin-Shephard questionnaire*, RSES: *Rosenberg Self-Esteem Scale*, PSWQ-C: *Penn State Worry Questionnaire for Children*, CISS:*Coping Inventory for Stressful Situations*, TiC-P: *Trimbos/iMTA questionnaire for Costs associated with Psychiatric Illness*.

### Randomization

Participants are randomly assigned to either the intervention or a waiting list. Randomization will take place at an individual level after the baseline measurement and one week before the start of the intervention. An independent researcher will make the allocation schedule with a computerized random number generator. The random allocation list will be generated in random permuted blocks of variable size (10, 12, 14, or 16). The randomization outcome per participant will be revealed to the primary investigator after the baseline measurement. In this manner, the investigator can give some background information (i.e., name, age, educational level) to the designated coach guiding the intervention participant through the course. Though the researcher has knowledge of participants' group assignment after the baseline measure, all other measures consist of automated online questionnaires and so there will be no contact here between participants and the primary researcher at all. Adolescents agree to participate before randomization and without knowing which group they will be allocated to. Received baseline questionnaires are numbered in order of arrival.

### Sample size

The sample size is based on the expected difference (Cohen's d = .50) between the intervention group and the wait-list control group at post-test, on the primary outcome variables, i.e., depressive and anxiety symptoms. Based on an alpha of .05 and a power of .80 in a two-tailed test, we need 63 subjects in each condition. Because Internet interventions tend to result in relatively high dropout rates of up to 40%, we aim for 210 participants. The expected effect size and dropout rate are derived from our experiences with an earlier study on the proposed intervention in adults [27]. High dropout rates for Internet self-help interventions are common though understandable when recognizing the multiple paths and trajectories of web usage [33]. Trials of Internet interventions attribute broad and unfiltered participant catchment, high and anonymous accessibility, ease of enrolment, and little personal or financial commitment [34].

### Interventions

#### Problem-Solving Treatment

The PST intervention is a Dutch adaptation of SET [28]. It has been expanded with more information, examples, and exercises. The theoretical assumption underpinning problem-solving therapy is that psychological symptoms of depression and anxiety are often caused by an inability to solve practical problems. Thus, symptoms will improve when problem-solving ability is enhanced.

PST in this study consists of several steps and takes five weeks, with one lesson a week. In the first lesson, subjects make a list of what is most important in their lives (e.g., my parents, my friends, school) and a list of their current worries and problems. Having listed their problems, participants subsequently divide these into three categories: (a) unimportant problems (problems unrelated to the things that matter to them), (b) important problems which can be solved, and (c) important problems which cannot be solved (e.g., the loss of a loved one). In the following weeks, participants can adapt their "important things" and "problem" lists while they learn to deal with the three types of problem introduced in the first lesson.

In lesson 2, subjects are taught the focal component of the intervention which is a specific six-step problem-solving procedure for structurally resolving important problems which can be solved. In the first step of this procedure, participants need to describe their problem. The next steps involve (2) writing down all possible solutions you can think of, (3) choosing the best solution, (4) describing how the solution will be carried out (when, with whom, where), (5) carrying out your plan of the solution, and (6) checking whether the problem is resolved. Participants practice with this six-step PST procedure from lesson 2 onwards; all other material and exercises only play a supporting role.

Lesson 3 deals with problems unrelated to things which are important, by proposing different strategies to eliminate negative thoughts and enhance positive thoughts. Strategies to eliminate negative thoughts concern (a) establishing one or two 15-minutes sessions a day for thinking about a problem (b) forcing yourself to immediately stop negative thoughts when they pop up, and (c) distracting yourself when ruminating, e.g., by calling a friend or engaging in sports. Two exercises for positive thinking are proposed; these are (a) thinking about three things which gave you a good feeling that day, and doing this every night before going to sleep, and (b) writing as many positive thoughts (things that made you happy, things that you are proud of) as possible on small cards, and then pulling one of these out regularly.

In lesson 4 - about important problems which cannot be solved -, participants are told that there are no rules for coping with a major event, but that it usually helps not to avoid negative emotions. Avoidance can be reduced by writing about your feelings, talking about your experiences with people that you feel close to, or by getting in touch with fellow-sufferers. Links to Dutch websites for specific types of problems are provided on the website.

During the fifth and last lesson, subjects look at their "problem list" again and are encouraged to identify the most important problem area - the problem most closely related to their feelings of depression and/or anxiety. They subsequently write down their goals concerning this problem for the long term. What are your goals for the next four months, and what are you going to do when encountering difficulties? Subjects also look at their "important things list" and are encouraged to think about actions they can undertake in order to reach these important things. Other exercises include making plans of how to deal with upcoming important life events which might make you feel low, and making a plan of what to do when encountering signals of depression or anxiety again; i.e., what steps they can take in order to prevent a relapse.

**Figure 2:**
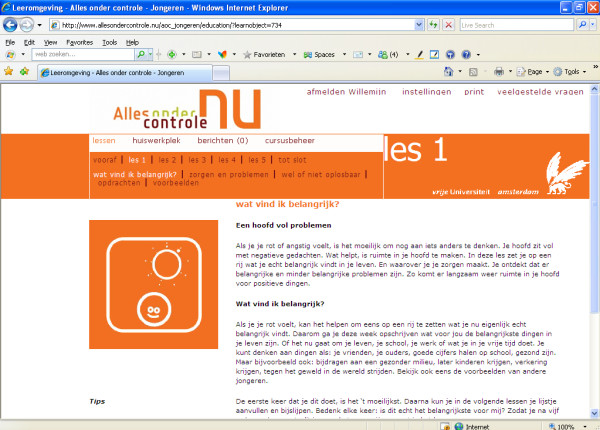
**Printscreen website**.

After the last lesson the website provides some general tips for counteracting depressed and anxious feelings, and phone numbers of professional mental health institutes are supplied. Figure 2 shows a typical website page.

#### Support

Subjects in the intervention group receive email support from employees of the Prevention and Intervention group of a mental health care institute in Amsterdam and the first two authors of this paper. Support is directed at guiding the participant through the intervention. This is done by sending an email if the coach does not receive participants' exercises at the end of the lesson and by commenting on the exercises made by participants. Comments include thanking the participant for sending and completing the exercises, compliments about exercises done well, asking for clarification when necessary, answering questions participants might have about the exercises, and giving tips when finding that the participant did not fully understand the exercise (e.g., "next time, I would like you to go into a bit more detail"). When participants send their assignments to their coach, they receive feedback within three working days. Support is not intended to give direct or individual advice on how to cope with depression, anxiety or other problems. Although performing the course with email support is strongly recommended, participants are permitted to complete the intervention without support.

#### Wait-list control group

Participants on the waiting list receive no intervention or support, only a link to a website with general information about depression and anxiety. They can commence the intervention four months after the intervention group starts the course.

### Instruments

Screening measures, primary outcome measures, secondary outcome measures, measures of clinical predictors, and measures of mediating variables can be distinguished in this study. The instruments include a diagnostic interview by phone and self-report questionnaires which are filled in by participants through the Internet.

### Screening measures

A structured diagnostic interview and self-report questionnaires including suicidal ideation, depression, and anxiety are administered as screening measures in this study.

#### Diagnosis

The Diagnostic Interview Schedule for Children (version NIMH DISC-4.0) is a reliable and valid structured diagnostic interview designed for lay interviewers, which includes algorithms to diagnose DSM-IV disorders in children and adolescents [35]. Participants complete a telephone version of the generalized anxiety disorder, social phobia, panic, agoraphobia, major depression, and dysthymia modules of the NIMH DISC-4.0. Telephone versions of structured psychiatric interviews in both adults [36] and youth [37] have been found to have a high correlation with in-person interviews. All interviewers receive 12 hours of training on the administration of the NIMH DISC-4.0. Meeting diagnostic criteria for a depression or anxiety disorder is not used as an exclusion criterion.

#### Suicidal ideation

The Beck Depression Inventory II (BDI-II) [38] measures the severity of self-reported depression in adolescents and adults. The time frame for the BDI-II ratings is for the "past two weeks, including today". The BDI-II has been found to encompass good psychometric properties. To screen for possible suicidal thoughts and intentions, the current study administered the Dutch suicide item of the BDI-II with scores of 0 ("I don't have any thoughts of harming myself"), 1 ("I have thoughts of harming myself, but I would not carry them out"), 2 ("I feel I would be better off dead"), and 3 ("I would kill myself if I could") [39]. Participants who score above the cut-off of 1 are excluded from the study.

### Primary outcome measures

Primary outcome measures include symptoms of depression as well as anxiety, because the intervention is intended to reduce symptoms of these two internalizing disorders. The questionnaires used for assessing these primary outcome measures, are also used as screening measures.

#### Depressive symptoms

The Centre for Epidemiological Studies Depression scale (CES-D) [40] is a widely used self-report measure for the screening of depressive symptoms in the week preceding the screening. It consists of 20 items for which subjects rate the frequency of symptoms during the past week with scores ranging from 0 (rarely or none of the time present [less than 1 day]) to 3 (most or all of the time present [5-7 days]), with a total score ranging between 0 and 60. Items represent major components of depressive symptomatology such as depressed mood, feelings of guilt and worthlessness, feelings of helplessness and hopelessness, psychomotor retardation, loss of appetite, and sleep disturbance. Though arbitrary, we looked at previous studies for mean CES-D scores and its standard deviation, and classified depressive symptoms as severe if they fall two standard deviations above the mean depression score. In this manner and based on these earlier studies [41,42], a score of 41 or higher on the CES-D means exclusion from this study. The validity of the CES-D has been tested in different populations [43-45], including studies with adolescents [41,42,46].

#### Anxiety symptoms

The Hospital Anxiety and Depression Scale (HADS) [47] is an extensively used, brief self-report screening scale to investigate the prevalence of depression and anxiety symptoms. The anxiety subscale of the HADS (the HADS-A) is used for the assessment of anxiety symptoms in this study. This subscale consists of 7 items rated on a four-point scale ranging from 0 (not at all) to 3 (a great deal of the time), with 3 indicating higher symptom frequency. A total score ranges from 0 to 21, and can be categorized as: normal (0-7), mild (8-10), moderate (11-14), or severe (15-21). To exclude adolescents with possible severe anxiety symptoms, a cut-off of 14 is used. The HADS shows good homogeneity and reliability, with Cronbach's alpha ranging from .81 to .84 in different normal and clinical Dutch samples [48], and has been found valid and adequate for use with adolescents [49].

### Secondary outcome measures

Secondary outcome measures include quality of life, symptoms of social anxiety, and cost-effectiveness.

#### Quality of life

The Pediatric Quality of Life Inventory 4.0 (PedsQL) is a self-administered paper-and-pencil questionnaire designed to assess quality of life in children and adolescents [50]. It includes parallel child self-reports (age range 5-18 years) and parent/carer proxy reports (age range 2-18 years). The 23-item self-report measure consists of four subscales: Physical Functioning (PH), Emotional Functioning (EM), Social Functioning (SOC), and School Functioning (SCH). In the present study, the PH, SOC, and SCH subscales of the PedsQL 4.0 child self-report for ages 12-18 are administered. Responses are given on a five-point scale ranging from 0 (never a problem) to 4 (almost always a problem). The Dutch PedsQL 4.0 shows good reliability and validity [51].

#### Social anxiety

The Social Anxiety Scale for Adolescents (SAS-A) [52] is one of the most widely used questionnaires for measuring social anxiety. This self-report scale consists of 18 anxiety-related items and four filler items assessing social preferences or activities. Each item is rated on a 5-point Likert scale according to how much the item "is true for you", ranging from 1 (not at all) to 5 (all the time). The SAS-A includes three subscales: Fear of Negative Evaluation (FNE; 8 items), Social Avoidance and Distress Specific to New Situations (SAS-New; 6 items), and Generalized Social Avoidance and Distress (SAS-General; 4 items). Scores from the three subscales are summed to form a total score. The SAS-A scales are found to have good internal consistency and adequate test-retest reliability [53]. For the current study, fears, concerns, and worries regarding negative evaluations from peers will be assessed using the FNE subscale of the Dutch version [54] of the SAS-A.

#### Direct and indirect costs

The Trimbos and iMTA questionnaire on Costs associated with Psychiatric Illness (TiC-P) [55] is used to collect data on direct and indirect costs. Direct costs are defined as the monetary valuation of the resources used to detect and treat medical problems. Indirect costs are defined as the productivity lost due to absenteeism and reduced efficiency at work or school. The first part of the TiC-P consists of questions on the number of contacts with health care providers. Next, health-related school absenteeism and failure to participate in sports are assessed.

### Measurement of predictors

Predictors that might distinguish adolescents who benefit from the intervention, include demographic variables, motivation, treatment credibility and expectancy, externalizing behaviour, perceived social support from significant others, substance use, the experience of life events, physical activity, the quality of the therapeutic alliance, and satisfaction.

#### Demographic variables

A self-designed demographic questionnaire is used to collect participants' demographic information. This instrument consists of 15 questions concerning nationality, ethnic origin, living situation, and education.

#### Motivation

A self-designed questionnaire is used to assess participants' willingness to spend time on the intervention. The instrument consists of 5 questions rated on a 5-point Likert scale ranging from "I totally disagree" to "I totally agree".

#### Treatment credibility and expectancy

The Credibility/Expectancy Questionnaire (CEQ) [56] assesses how believable, convincing, and logical a particular treatment seems to the patient, and the personal improvements the patient believes will be achieved. The questionnaire consists of six items and uses two rating scales, one from 1 (not at all) to 9 (very much) and another from 0% (not at all) to 100% (very much). The CEQ showed high internal consistency and good test-retest reliability [56].

#### Externalizing behaviour

The Youth Self Report (YSR) [57] is a 101-item self-report questionnaire measuring problem behaviours in adolescents aged 11-18 years. Adolescents are asked if they have experienced certain problems in the preceding 6 months, and the response options are "not present", "somewhat or sometimes true", or "very true or often true". The YSR provides eight subscales. To measure externalizing behaviour, the delinquent and aggressive behaviour subscales encompassing the externalizing problem scale of the YSR are administered (Dutch version; [58]). To shorten this original 30-item problem scale, items having factor loadings < .40 in one of our datasets were omitted, leaving us with 6 items on delinquent behaviour, *α *= .57, and 10 items on aggressive behaviour, *α *= .75. This short version of the YSR externalizing scale has an alpha of .79. Good reliability and validity estimates of the YSR have been documented [57].

#### Perceived social support

The Social Support Scale for Adolescents (SSSA) [59] is a 24-item self-report measure assessing adolescents' perceived social support from significant others in their life, including parents, teachers, classmates, and close friends. The SSSA assesses the degree to which adolescents perceive that others care for them as individuals, like them the way they are, understand them, listen to them and generally treat them as people who matter. The "parents" and "close friends" subscales are administered in this study. Both scales consist of six items scored on a four-point scale, with higher scores indicating greater perceived support. Harter [59] reported good reliability and validity of the SSSA.

#### Substance use

##### Alcohol use

Adolescents are asked to respond to two questions about (1) how often they had consumed alcohol in the past 4 weeks, and (2) the number of occasions on which seven or more drinks in a row were consumed in the past 4 weeks. Answers are rated on a 6-point scale (1 = did not drink any alcohol in the past 4 weeks; 2 = drank alcohol at 1 to 3 days in the past 4 weeks; 3 = drank alcohol at 1 to 2 days per week; 4 = drank alcohol at 3-4 days per week; 5 = drank alcohol at 5-6 days per week; 6 = drank alcohol every day in the past 4 weeks).

##### Smoking

Smoking behaviour is assessed with the question "Have you ever smoked even part of a cigarette?" Current smokers are those who mark the response "yes, I now smoke cigarettes". Non-smokers are those who mark any other response option, ranging from "No, I've never smoked even part of a cigarette" to "Yes, I used to smoke at least once a week, but I quit". Daily smoking is assessed with the question "how much on average do you smoke per day?" Response options range from 0 (less than one cigarette per day) to 6 (more than 30 cigarettes per day).

##### Drug use

Drug use is measured by asking participants to indicate how often, if ever, they have used soft drugs in the last twelve months. This question is also posed for using hard drugs. Responses range from 0 (never) to 13 (40 times or more).

#### Life events

Adolescents complete a 12-item short form of the Life Event Questionnaire [60], which is a yes-or-no format self-report questionnaire assessing potentially stressful life events such as parental divorce, death of a family member, or long-term hospitalization in the past two years. The item scores are summed into a total life event score, with higher scores indicating more life events. The test-retest reliability of the Dutch LEQ for the total life event score was reported to be .90 [60].

#### Physical activity

Physical activity is measured with the Godin-Shephard questionnaire [61]. This questionnaire measures the habitual number of activities per week at various levels of intensity: light (e.g., walking), moderate (e.g., badminton), and strenuous (e.g., basketball). A total physical activity score is calculated. The scale has been validated for children and adolescents [61,62].

#### Working alliance

The Working Alliance Inventory (WAI) is a measure of the quality of the therapeutic alliance between the client and therapist. The original 36 items of this self-report questionnaire are rated on a 7-point Likert scale and measure three distinct factors of the therapeutic relationship: the therapeutic bond, task agreement, and agreement on therapeutic goals. Good psychometric properties have been found [63]. For this study, the 12-item short form of the WAI is used.

#### Client satisfaction

The 8-item Client Satisfaction Questionnaire (CSQ-8) is a one-dimensional instrument to assess global patient satisfaction [64]. This shorter version of the original 18-item scale had the same construct validity and internal consistency reliability as the longer version [64]. The CSQ-8 items can be scored on a scale from 1 to 4 with a total score ranging from 8 to 32.

### Measures of mediating variables

To test whether the basic components of the intervention mediate the effects of the treatment on changes in depressive and anxiety symptoms, questionnaires including problem-solving skills, worrying, mastery, and self-esteem are administered.

#### Problem-solving skills

The Coping Inventory for Stressful Situations (CISS) [65] is a 48-item self-report measure composed of three scales assessing problem-focused behaviours, emotion-focused behaviours, and avoidance strategies. Problem-solving ability is measured with the subscale "task oriented coping" (problem-focused strategies) of the CISS. This subscale consists of 16 items scored on a five-point Likert scale, referring to the extent to which people make use of problem-solving techniques in the face of stress, with answers ranging from "not at all" to "very strongly". Scores range from 16 to 18. The CISS has a stable factor structure, excellent internal consistency, and adequate test-retest reliability [65,66].

#### Worrying

The tendency of adolescents to engage in excessive, generalized, and uncontrollable worry is assessed with the Penn State Worry Questionnaire for Children (PSWQ-C) [67]. The PSWQ-C consists of 14 items, which are scored on a 4-point scale varying from "not at all true" to "always true". The PSWQ-C possesses good reliability and validity estimates [67].

#### Mastery

Perceived control is assessed with the Mastery Scale [68]. The seven items on the scale measure the extent to which participants see themselves as being in control of the forces that significantly affect their lives. Responses are rated on a 5-point Likert scale ranging from "strongly disagree" to "strongly agree". The items are summed for a total mastery/competency score. The questionnaire has good psychometric properties [68].

#### Self-esteem

Self-esteem is measured with the Rosenberg Self-Esteem Scale (RSES) [69], a widely-used measure of global self-esteem in adolescents. The scale consists of 10 items of positive and negative aspects of self-esteem, and is scored as a 4-point Likert scale, with responses ranging from "strongly agree" to "strongly disagree", yielding scores between 10 and 40. The scale shows good psychometric properties [70].

### Statistical analysis

Intention-to-treat and completer analyses will be performed. Overall, treatment efficacy will be assessed with linear mixed modelling analysis using SPSS. For analyzing mediating variables and for the identification of subgroups in the sample, general growth mixture modelling will be applied, using M-*plus*. With this method, it is possible to identify distinct groups of individuals, differing in the initial level and course of a specific behaviour, through the empirical identification of developmental trajectories [71]. This technique also makes it possible to examine whether the effects of an intervention differ for various categories of subjects, and to determine which characteristics (moderators) predict membership of one of these categories [72].

## Discussion

This study compares a preventive problem-solving guided self-help intervention through the Internet with a wait-list control group and aims to provide insight into the efficacy of the Internet-based intervention for adolescents. A secondary objective is to examine how the intervention works and for whom. A discussion of specific strengths and limitations of this study follows below.

First of all, a strength of this study is that it is a practice-based project and both research aims relate to important matters in the treatment of adolescents with symptoms of depression and anxiety. There is a lack of studies on the efficacy of preventive self-help interventions for adolescents with emotional problems, which limits the evidence base for this treatment method. Simultaneously, insight into the questions as to which subgroups respond differently to the intervention and why and how the intervention led to change is scarce. Mechanisms of change are rarely studied in child and adolescent therapy, though the study of mechanisms of treatment can serve as a basis for maximizing treatment effects and ensuring that critical features are generalized to clinical practice [73]. Results of this study offer encouragement with regard to the implementation of an effective self-help Internet intervention for reducing depressive and anxiety symptoms in adolescents and preventing or postponing the onset of depression and anxiety disorders.

A strength of our intervention in particular is that it is offered through the Internet; it constitutes a self-help format, and may be used in adolescents with different types of comorbid problems. This is especially salient since a large group of untreated adolescents can therefore be reached.

A strong aspect of the design of this study is the number of measurements. Six measurements are used, making it possible to analyze the role of potential mediating variables in predicting intervention effects and the development of different kind of symptoms over time.

Another advantage of this study concerns the possibility to compare results with studies using clinical samples. Though subjects are included on the basis of self-rating instruments - as the intervention is intended to be applicable and accessible for a broad population with self-reported mild to moderate depressive and anxiety symptoms - information about whether subjects meet criteria for Major Depression, Dysthymia, Panic, Agoraphobia, Social phobia, and Generalized Anxiety Disorder is assessed. The standardized diagnostic interview is not used at posttest, however, so this study does not examine whether the intervention is actually capable of reducing the incidence of cases of depression and anxiety as defined by diagnostic criteria. When using a diagnostic interview both at baseline and follow-up, large numbers of subjects are needed to yield sufficient statistical power to be able to show significant effects on incidence [74]. Moreover, seeing that recruitment and drop-out are major issues in adolescent studies, we wanted to keep the threshold for participating as low as possible, without losing vital information.

A limitation of this study includes the relatively small sample size, making it difficult to draw firm conclusions about the moderation and mediation research questions. As our trial is primarily focused on determining whether the Internet intervention is a feasible and effective preventive intervention for adolescents with subsyndromal anxiety and depression, power is only calculated for our primary outcome measures. With regard to moderating and mediating variables or the effectiveness of our intervention for specific subgroups, our study is of an explorative nature, which will permit us with enough power to detect rather robust effects, while other less prominent associations may be more difficult to discern.

Another limitation and expected problem constitutes refusals to participate in this study. Due to ethical considerations, only adolescents who are willing to ask for their parents' consent to participate in the current trial can be included. However, adolescents would often prefer to participate without parental consent. Negative parent-child relationships were also found to be related to depression in adolescence [12], suggesting that a considerable percentage of adolescents with emotional complaints also have problems at home, making it more difficult to inform their parents about the study. Recruitment of participants might thus be difficult, and characteristics of adolescents who ask their parents' consent for study participation might be different from adolescents who do not ask for permission. At the same time and as reported in many studies [75], characteristics of adolescents whose parents give consent compared to adolescents whose parents do not, might be different. This may lead to selection bias, and the results may not be generalisable to all adolescents with depressive and anxiety symptoms. On the other hand, characteristics of participants can be compared to depressed or anxious adolescents who participate in non-research web-based interventions, in which parental consent is not required. Moreover, requesting parental consent might be to our advantage, as ease of dropout is reduced, which makes a lower dropout rate likely.

In conclusion, many adolescents report symptoms of depression and anxiety but do not seek help in regular healthcare. This study aims to contribute to the evidence-based preventive treatment of emotional problems in adolescents by investigating problem-solving self-help therapy via Internet.

## Competing interests

The authors declare that they have no competing interests.

## Authors' contributions

PC and HMK obtained funding for the study. All authors contributed to the design of the study and the adaptation of the Internet-based PST intervention. WH coordinates the recruitment of participants and data collection during the study. JS and WH supervise the Problem-Solving Therapy. WH wrote the manuscript. All authors contributed to the further writing of the manuscript. All authors read and approved the final manuscript.
